# A Robust Design Capture-Recapture Analysis of Abundance, Survival and Temporary Emigration of Three Odontocete Species in the Gulf of Corinth, Greece

**DOI:** 10.1371/journal.pone.0166650

**Published:** 2016-12-07

**Authors:** Nina Luisa Santostasi, Silvia Bonizzoni, Giovanni Bearzi, Lavinia Eddy, Olivier Gimenez

**Affiliations:** 1 Dolphin Biology and Conservation, Oria, Italy; 2 Centre d'Ecologie Fonctionnelle et Evolutive, Montpellier, France; 3 OceanCare, Wädenswil, Switzerland; 4 Sapienza Università di Roma, Roma, Italy; University of Missouri Kansas City, UNITED STATES

## Abstract

While the Mediterranean Sea has been designated as a Global Biodiversity Hotspot, assessments of cetacean population abundance are lacking for large portions of the region, particularly in the southern and eastern basins. The challenges and costs of obtaining the necessary data often result in absent or poor abundance information. We applied capture-recapture models to estimate abundance, survival and temporary emigration of odontocete populations within a 2,400 km^2^ semi-enclosed Mediterranean bay, the Gulf of Corinth. Boat surveys were conducted in 2011–2015 to collect photo-identification data on striped dolphins *Stenella coeruleoalba*, short-beaked common dolphins *Delphinus delphis* (always found together with striped dolphins in mixed groups) and common bottlenose dolphins *Tursiops truncatus*, totaling 1,873 h of tracking. After grading images for quality and marking distinctiveness, 23,995 high-quality photos were included in a striped and common dolphin catalog, and 2,472 in a bottlenose dolphin catalog. The proportions of striped and common dolphins were calculated from the photographic sample and used to scale capture-recapture estimates. Best-fitting robust design capture-recapture models denoted no temporary emigration between years for striped and common dolphins, and random temporary emigration for bottlenose dolphins, suggesting different residency patterns in agreement with previous studies. Average estimated abundance over the five years was 1,331 (95% CI 1,122–1,578) striped dolphins, 22 (16–32) common dolphins, 55 (36–84) “intermediate” animals (potential striped x common dolphin hybrids) and 38 (32–46) bottlenose dolphins. Apparent survival was constant for striped, common and intermediate dolphins (0.94, 95% CI 0.92–0.96) and year-dependent for bottlenose dolphins (an average of 0.85, 95% CI 0.76–0.95). Our work underlines the importance of long-term monitoring to contribute reliable baseline information that can help assess the conservation status of wildlife populations.

## Introduction

The need for preserving cetaceans in the Mediterranean Sea is recognized in several international agreements (e.g. ACCOBAMS, the Agreement on the Conservation of Cetaceans of the Black Sea, Mediterranean Sea and Contiguous Atlantic Area) and robust assessments of population abundance, trends and distribution are necessary to inform conservation actions [1]. Obtaining such quantitative information about cetacean populations, however, is expensive and logistically challenging [2]. The evaluation of the conservation status of Mediterranean cetaceans has been hampered by poor information for all cetacean species, especially in the southern and eastern portions of the region [3]. Here, we partially fill this gap by providing detailed quantitative information on the abundance of three cetacean species inhabiting the Gulf of Corinth in Greece: the striped dolphin (*Stenella coeruleoalba*), the short-beaked common dolphin (*Delphinus delphis;* hereafter “common dolphin”) and the common bottlenose dolphin (*Tursiops truncatus;* hereafter “bottlenose dolphin”).

The Gulf of Corinth is a semi-enclosed basin located between continental Greece and the Peloponnese. It contains a variety of pelagic and coastal habitats within a relatively restricted area (2,400 km^2^) and hosts a unique mixture of sympatric pelagic and coastal odontocetes [4]. Because of its relevance for cetaceans, the Scientific Committee of ACCOBAMS listed the Gulf of Corinth as an area of special conservation importance and called for the creation of a marine protected area (Resolution 3.22; [5]). In this area, striped dolphins are the most abundant cetacean species [4, 6]. Common dolphins are found only in mixed-species groups with striped dolphins, and individuals showing an intermediate pigmentation suggest the occurrence of hybridization [4, 6]. Bottlenose dolphins also occur in the Gulf in single-species groups.

**Fig 1:**
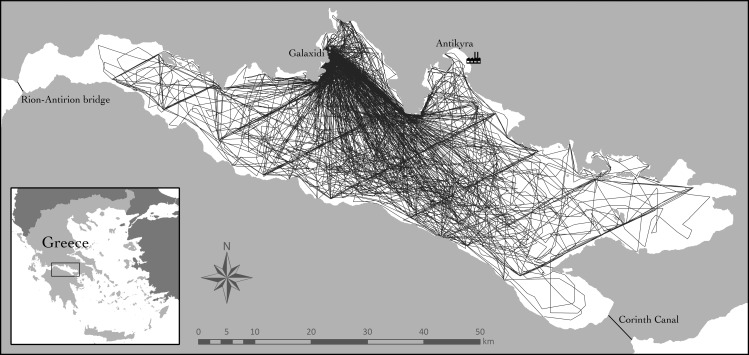
The Gulf of Corinth study area in Greece, with survey tracks in 2011–2015. The locations shown in the map include: Galaxidi (our base port), the aluminum factory (indicated by an icon) near Antykira, the artificial Corinth Canal that connects the Gulf to the Aegean Sea and the Strait of Rion (crossed by the Rion-Antirion bridge) that connects the Gulf with the Ionian Sea.

This semi-enclosed area is vulnerable to a number of human impacts that affect coastal areas throughout the Mediterranean [7]. The main known anthropogenic impact in the Gulf is an industry for aluminum production that has been operating in the Bay of Antikyra since 1966 (Fig 1), dumping massive quantities of industrial discards in the Gulf's waters [8, 9, 10]. The impact of overfishing remains scarcely documented, but likely to have caused significant ecosystem change in the Gulf of Corinth, as reported from other parts of the Ionian Sea [11].

While global populations of striped, common and bottlenose dolphins are classified as Least Concern in the IUCN Red List of Threatened Species [12, 13, 14], the Mediterranean “subpopulation” (*sensu* IUCN) of common dolphins is classified as Endangered [15], whereas striped and bottlenose dolphins are Vulnerable [16, 17]. Because of their restricted range and their apparent geographic [4, 6, 18] and reproductive isolation [19, 20, 21, 22], striped and common dolphins in the Gulf of Corinth are vulnerable to local anthropogenic pressures such as habitat degradation, prey depletion and anthropogenic noise [23, 24, 25]. It is therefore important to monitor their status and abundance. Even though bottlenose dolphins are known to perform mid-distance movements to areas outside of the Gulf of Corinth [26], knowing how many individuals use this area on a regular basis is important to evaluate their conservation status and propose management actions at the local scale.

In this context, the lack of robust abundance baselines may prevent the detection of decline resulting from anthropogenic impacts [27, 28, 29]. Here, we contribute robust estimates of abundance and survival that can be used to inform conservation action in the Gulf of Corinth. To do so, we apply capture-recapture (CR) methods [30, 31, 32] that have been used extensively on several taxa (reviewed by [33]) including marine mammals [34, 35, 36, 37, 38, 39, 40, 41]. Several cetacean species can be individually photo-identified based on long-term natural markings, allowing for the application of non-invasive CR methods [42]. Two families of CR models can be used to estimate abundance, depending on the duration of the sampling period and the movement patterns of the studied population. Closed population models rely on the assumption of population closure to additions (births or immigration) and losses (death or emigration) for the duration of the study [31]. This assumption can be relaxed using open population models that allow for entries and losses [43, 44]. However, these models do not fully accommodate for multiple movements in or out of the study area (so-called temporary emigration; e.g. [45]).

Pollock’s robust design (RD) [46, 47, 48] offers an alternative approach. It has been applied on terrestrial animals e.g. [49, 50] and on a number of cetaceans species including common bottlenose dolphins e.g. [38], Indo-pacific bottlenose dolphins *Tursiops aduncus* [51, 52] and Guiana dolphins *Sotalia guianensis* e.g. [53]. It relies on a number of primary sampling occasions, each being composed of secondary occasions [46]. The time interval between secondary sampling occasions must be short enough to meet the population closure assumption, while consecutive primary occasions should be sufficiently separated in time to allow the population to change. Data from secondary samples within each primary period are analyzed using closed models to derive estimates of capture probability and population size. Apparent survival and temporary emigration are estimated using open models by collapsing data from the secondary periods. In general, RD estimates are more accurate and precise than those obtained through the application in sequence of closed and open models because they allow estimation of survival and abundance while accounting for temporary emigration [45, 48, 54].

In this study, our objectives were threefold. First, we addressed different degrees of site fidelity by carefully designing the sampling periods to meet RD model assumptions. Second, we applied a RD approach to striped, common and bottlenose dolphins in the Gulf of Corinth and estimated their abundance and survival in years 2011–2015. Third, we evaluated the statistical power of our monitoring program in detecting a potential temporal trend in abundance given the achieved level of survey effort, and provided suggestions for future monitoring and management [2, 55, 56]. Overall, our study contributes important baselines to assess dolphin conservation status and provides a methodological framework to investigate abundance under a challenging scenario including the occurrence of mixed-species groups, highly diverse population numbers, and contrasting site fidelity patterns.

## Materials and Methods

### Ethic statement

Data collection (individual photo-identification of free-ranging animals from boats) was done based on research permits issued by the Hellenic Ministry of Environment, Energy and Climate Change, in compliance with legal and ethical principles of animal welfare. Data collection entailed no handling of animals, no harm caused to animals, and no harassment of animals. Research permits issued by the Hellenic Ministry of Environment, Energy and Climate Change do not require further assessment by an animal ethics committee, and the benign research conducted for in the context of this study does not raise ethical issues.

### Study area

The Gulf of Corinth is a semi-enclosed basin located in the eastern Mediterranean Sea, between the Peloponnese and mainland Greece. It has a surface area of 2,400 km^2^, a length of about 130 km and a maximum width of about 32 km. It is connected to the Ionian Sea through the Strait of Rion (maximum width 2 km, maximum depth 65 m) and to the Aegean Sea through the artificial Corinth Canal (width 21 m, length 6.4 km, maximum depth 8 m) that crosses the Isthmus of Corinth. The edge of the northern continental shelf is characterized by gentle slopes, while the southern continental slope is steeper. The Gulf reaches a maximum depth of 935 m.

### Sampling methods

Navigation was conducted from a 5.8 m boat with a 100 HP four-stroke outboard engine, from May to October, between 2011 and 2015. The duration of the sampling season was determined largely by weather constraints and funding availability. One day of sampling was defined as a “survey” and each encounter with a dolphin group as a “sighting”. All surveys started and ended at the port of Galaxidi (Fig 1). Apart from this constraint, survey routes were intended to attain an extensive coverage of the study area during each month of sampling, with different area coverage in different days (also depending on sea state and weather conditions). Navigation in search for dolphins was carried out under the following conditions [6]: (1) daylight and long-distance visibility, (2) sea state ≤ 2 Douglas, (3) at least two experienced observers scanning the sea surface, (4) eye elevation of approximately 1.6–1.8 m for both observers, and (5) survey speed between 26 and 30 km/h. Binoculars were not used during navigation.

### Photo-identification

When a dolphin group was sighted we approached the group slowly to minimize disturbance. Individual photo-identification was conducted following [57]. We took photos from a maximum distance of about 20 m to a minimum distance of about 1 m when the animals voluntarily approached the boat. Photographs were taken using 18 megapixel SLR cameras equipped with 70–200 mm f2.8 zoom lenses. Photographic identification was used as a “capture” method [57]. Individual identification of dolphins relied on nicks and notches visible from both sides of the dorsal fin [58]. We attempted to photograph the dorsal fin of all dolphins in each encountered group, taking as many photos as possible of both dorsal fin sides. Photos were taken randomly, regardless of dorsal fin markings. Once a group was left, we either returned to the port or continued navigation in search for dolphins.

All photos taken in the field went through a first selection to exclude those without dolphins, those out of focus and those in which the dorsal fin was not completely visible. The remaining photos were imported in Adobe Lightroom and cropped around the dorsal fin and the visible part of the body. The correct identification of individuals is a fundamental assumption of CR methods [31]. To meet this assumption, we stratified our sample based on 1) photo quality categories (adapted from [34]), and 2) dorsal fin distinctiveness [35, 59]. All images were scored after being cropped around the dorsal fin. We assigned a quality score of 1 to 3 to each photo based on sharpness, exposure and angle of the dorsal fin. Partially obscured fins (e.g. by water spry or other dolphins) were discarded. Grade 3 and 2 photos were judged suitable for the recognition of marked fins and they also ensured recognition of small markings. Grade 1 photos were dropped because they were considered suitable only for the recognition of the most marked individuals. Additionally, we stratified the individuals into different distinctiveness categories corresponding to the degree of natural markings on the leading and trailing edges of the dorsal fin [60]. Three categories were used: D1 (multiple big or medium notches; distinctive features, which would be recognizable in distant or poor-quality photos); D2 (smaller nicks, which would not be recognizable in distant or poor-quality photos); and D3 (subtly marked or unmarked fins). Only individuals classified as D1 were included in the analyses. During matching, each distinctive dorsal fin photo was compared with all others and an identity code was assigned to photos of the same individual. Once the matching process was completed, three experienced operators re-checked the whole catalogue to look for false positive and false negative errors [59].

### Mixed-species groups

Because it was impossible to discriminate between striped, common and intermediate individuals based on dorsal fin photographs alone, striped and common dolphins as well as individuals with intermediate pigmentation were considered together in CR analyses [6]. The proportion of striped and common dolphins in the population was estimated based on a subset of photographs of animals showing relevant portions of their body during aerial behavior, or performing other conspicuous surfacings [6]. We included in the analyses exclusively well-lit photos cropped around visible portion of the body, portraying one side of the animal (ventral, dorsal, front and rear views excluded), including: 1) jumps, leaps or energetic surfacings exposing the whole body or at least three-fourths of it, including dorsal fin and whole lateral portion of the body; and 2) surfacings showing upper lateral portion of the body including whole dorsal fin, eye and upper flank. Retained images had 100% agreement between two independent assessors with 15+ years of experience. If either assessor was unable to attribute a species category the image was discarded. Following this filtering step, the final abundance estimate was corrected using the proportion of photographs of each species, and a coefficient of variation calculated for the abundance estimate of each species [6] as:

$$CV=\sqrt{{\left(CVspecies\right)}^{2}+{\left(CVdistinctiveness\right)}^{2}+{\left(CVN\right)}^{2}}$$

where *CVspecies* is the coefficient of variation of the proportion of the different species, *CVdistinctiveness* is the coefficient of variation of the proportion of marked individuals, and *CVN* is the coefficient of variation of the total population estimate.

### Capture-recapture matrices

We considered two separate datasets: one for bottlenose dolphins and one for striped, common and intermediate individuals. For each dataset, we built a capture matrix (i.e. a binary table with individuals in rows and sampling occasions in columns). The entries of the matrix are 1s if an individual is detected in a sampling occasion and 0s if the individual is not detected. A “capture” was defined as a photograph of sufficient quality of an individual dolphin’s distinctively marked dorsal fin, obtained during a sampling occasion. These matrices were used to estimate abundance with RD models [46, 47]. These models are based on primary and secondary sampling occasions: the population is assumed closed between secondary occasions and open between primary occasions. Schematically, data from primary occasions are used to estimate apparent survival and temporary emigration rates using open population models [43], whereas data from the secondary occasions are used to estimate population abundance using closed population models [31].

To meet the closed population assumption, we set primary and secondary occasions of different durations for the two species to account for their different movement patterns in and out of the study area (Table 1). Striped and common dolphins in the Gulf of Corinth are considered geographically isolated subpopulations [4, 6]. In this case, we considered annual sampling seasons (i.e. between May and October) as primary sampling occasions. We collapsed the sighting histories on a monthly basis using months as secondary sampling occasions, as they correspond to one monthly coverage of the entire study area.

**Table 1:** Primary and secondary capture occasions for the two capture matrices (striped and common dolphins, and bottlenose dolphins). All sampling days with encounters of the three species are listed in S1 and S2 Tables.

Striped and common dolphins					
Primary occasions	Summer 2011	Summer 2012	Summer 2013	Summer 2014	Summer 2015
Secondary occasions	12–30 May	7–10 June	6–23 June	7–30 June	4–30 June
	14–24 June	8–15 July	2–30 July	7–26 July	7–24 July
	2–16 July	5–25 August	10–24 August	5–31 August	1–29 August
		12–28 September	2–9 September	8–20 September	11–26 September
Bottlenose dolphins					
Primary occasions	15 June—8 July 2011	8–11 July 2012	8–11 July 2012	11–17 June 2014	6–14 July 2015
Secondary occasions	15 June	8 July	8 July	11 June	6 July
	20 June	10 July	9 July	13 June	13 July
	8 July	11 July	10 July	16 June	14 July
			11 July	17 June	

Because bottlenose dolphins in the Gulf of Corinth are known to perform mid-distance movements to areas outside of the Gulf [26], we considered a short time frame for secondary occasions, consistent with the assumption that emigration did not occur within those periods. We selected primary occasions with the aim of maximizing sample size while minimizing the duration to reduce the risks of violating the closure assumption. Primary occasions for bottlenose dolphins were intervals between 4 and 9 days long in June and/or July of each year of the study, with the exception of year 2011 due to reduced sample size. Secondary occasions were single days (Table 1).

### Estimating population parameters of distinctly marked individuals

RD models allow estimating population abundance, capture probability and apparent survival while accounting for temporary emigration [46, 47]. We define the temporary emigration parameter (ϒ”) as the probability of an individual being a temporary emigrant, given it was alive and present in the study area in the previous primary sampling occasion [47]. The other temporary emigration parameter (ϒ’) is the probability of an individual being a temporary emigrant given it was a temporary emigrant in the previous sampling occasion [47]. Apparent survival rate (S) is the probability of surviving and staying in the study area, and is the product of true survival and fidelity to the study area, while p is the capture probability [43, 46, 47, 48].

A set of 30 models composed of parameters for population size (N), apparent survival rate (S), temporary migration rates (ϒ”, ϒ’) and capture probability (p) were fitted to the data with program R [61], package RMark [62], to construct models from program MARK [63]. The following three temporary emigration patterns were considered: 1) no temporary emigration (ϒ” = ϒ’ = 0); 2) random temporary emigration, (ϒ” = ϒ’) where the probability of an individual being present in the study area is not dependent on whether or not it was present in the study area in the previous sampling period; and 3) Markovian temporary emigration (ϒ”,ϒ’) where the probability of an individual being present in the study area is conditional on whether it was present in the study area before. For all three patterns of temporary emigration, we considered models where apparent survival was either constant or varying between primary occasions and capture probability was either constant, or varying with time (between secondary occasions, between primary occasions, or both). The Akaike’s Information Criterion with a correction for small sample sizes (AICc) was used as a measure of relative model fit. The model with the lowest AICc was selected as the most parsimonious and parameter estimates were averaged when there were models within 2ΔAICc from the best model [64]. To explore the effect of heterogeneity in capture probabilities, we fitted our models with two-class finite mixtures [65] allowing for detection probabilities to vary among individuals (Mh) and among individual and secondary capture occasions (Mth). Under the two-class finite mixtures, individuals may belong to one class of animals with a capture probability p_1_ in some proportion π or to another class of animals with a capture probability p_2_ in proportion 1 –π.

### Validation of model assumptions

An important step when fitting CR models is to evaluate the validity of the assumptions underlying their construction [43]. Here we describe how we designed our study to meet each of the RD protocol assumptions. 1) *Individual marks are correctly recognized*: only high-quality photographs (Q3 and Q2) and highly marked (D1) dorsal fin markings were used to identify individuals and investigators with extensive experience double-checked all matches. 2) *The sampling interval for a particular secondary sample is instantaneous*: the sampling occasions selected for our analyses were relatively short in duration (1 week or 1 month) compared with the study period [36, 66]. 3) *The population is closed within primary periods*: genetic [19, 20, 21, 22] and distribution data [4, 6, 18] indicate that striped and common dolphin populations in the Gulf of Corinth are geographically isolated. For bottlenose dolphins we restricted the length of primary sampling occasions to 4–9 days so that movements could be negligible. A limited number of deaths might have occurred within each primary period. Because striped common and bottlenose dolphins are long-lived mammals with high survival rates [67], we considered as negligible the number of deaths that may have occurred within the duration of primary occasions (up to four months). Calves were all unmarked (distinctiveness category D3) and therefore they were not included in the estimation of D1 individuals. 4) *Capture and survival probability do not vary among individuals*. We tested these assumptions by i) fitting models incorporating heterogeneity in capture probability and ii) by running specific tests using program U-CARE [68] on the data pooled by primary occasions. TEST 2 evaluates the assumption of homogeneous detection probabilities and has two components. TEST 2.CT is interpreted as a test for trap dependence and tests the null hypothesis that individuals encountered and not encountered at occasion t have the same probability of being re-encountered at time t+1, conditional on their presence on both occasions. TEST 2.CL tests the null hypothesis that there is no difference in the expected time of next re-encounter between individuals encountered and not encountered at occasion t, conditional on their presence on occasion t and t+2, [43]. TEST 3 evaluates the assumption of homogeneous survival probabilities and has also two components. TEST 3.SR is interpreted as a test for transience and tests the null hypothesis that there is no difference in the probability of being re-encountered between “new” (never encountered before) and “old” (already encountered) individuals. TEST 3.Sm tests the null hypothesis that there is no difference in the expected time of first re-encounter between the “new” and “old” individuals encountered at occasion t and later re-encountered [43]. To date both TEST 2.CL and TEST 3.Sm have received no simple biological interpretation [69]. The global test (TEST 2 + TEST 3) is used as a goodness of fit test for the Cormack-Jolly-Seber model [43]. Whenever these tests were found significant, we calculated the variance inflation factor (ĉ; χ^2^ of the GOF test divided by the degrees of freedom) to account for the lack of fit [43].

### Population abundance

To estimate total population size, abundance of the marked population has to be corrected for the proportion of identifiable individuals. This proportion (θ) was calculated on an annual basis from the photographic sample as the ratio of high quality photographs (Q2 and Q3) with distinctive dorsal fins (D1) to the total number of Q2 and Q3 photos with distinctive and non-distinctive dorsal fins [70]. The total population abundance was then estimated as:

Ntot=Nm/θ

where *Ntot* is the estimated population size, *Nm* the estimated marked population size and θ the estimated mark ratio in the population. The variance of the total population estimate was calculated as:

$$Var(Ntot)={\left(Ntot\right)}^{2}\left(\frac{\mathrm{var}(Nm)}{{\left(Nm\right)}^{2}}+\frac{1-\theta }{n\theta }\right)$$

where *n* is the number of identified animals from which θ was estimated, *Nm* the estimated number of marked animals, θ the mark ratio and *Var(Nm)* the variance of *Nm* [34]. Log-normal 95% confidence intervals were calculated with a lower limit of *Ntotal/C* and an upper limit of *Ntotal x C* [34, 71], where *C* was calculated as:

$$C=\exp\left(1.96\sqrt{\ln\left(1+\left({\left(\frac{SE(Ntotal)}{Ntotal}\right)}^{2}\right)\right)}\right)$$

### Trends in abundance

To determine the ability of our monitoring to detect a population trend in abundance using linear regression, we performed a statistical power analysis [55]. A trend was detected when the regression of population abundance estimates over time had a slope significantly different from zero [55]. The conclusion that a trend in abundance is occurring when it is not, is called a Type 1 error (α). The conclusion that no trend in abundance is occurring when in fact it is, is a Type 2 error (β). The statistical power is expressed as 1 –β and is the probability of correctly detecting a trend using linear regression when it actually occurs. The power is related to the number of samples (n), the precision of the estimates (CV), the rate of change in the population (R) and the probability of Type 1 and Type 2 errors (α and β). We used a power analysis to calculate the minimum rate of change (R) that we were able to detect with an acceptable statistical power of 0.8 [56] given the duration of our monitoring plan (5 yearly samples) and the precision of our estimates. Analyses were conducted using the package “fishmethods” [72], setting the parameters as follows: the probability of Type 1 error α = 0.05, a linear type of change in abundance and a one-tailed test. For CR estimates the CV is expected to be proportional to the square root of abundance [56]. We calculated the overall CV of the monitoring period by averaging the annual CVs of estimates [53, 73].

## Results

### Sampling results

From 2011 to 2015 navigation was performed on 211 days and covered 21,435 km, yielding a total of 468 sightings of striped dolphins or mixed-species groups including striped dolphins, common dolphins, or animals of intermediate pigmentation, and 53 sightings of bottlenose dolphins. A summary of the sampling effort and photo-identification results by year is presented in Table 2.

**Table 2:** Main characteristics of the dataset. Survey effort, hours of dolphin tracking, number (#) of sampled groups, number (#) of high quality photos (Q2 and Q3) and percentage (%) of new identified individuals by year and by species (Sc+Dd = striped and common dolphins; Tt = bottlenose dolphins) are reported.

Year	survey effort(km)	hours of dolphin tracking		# of groups		# of Q2 and Q3 photos		% of new D1 individuals	
		Sc+Dd	Tt	Sc+Dd	Tt	Sc+Dd	Tt	Sc+Dd	Tt
2011	4,171	316	17	96	7	3,527	83	100	100
2012	3,362	342	53	77	10	4,570	462	39	83
2013	4,243	450	84	78	9	6,000	441	13	10
2014	4,514	382	104	100	15	4,817	1,038	12	12
2015	5,145	383	77	75	10	5,081	448	9	31
TOT	21,435	1,873	335	426	51	23,995	2,472		

**Fig 2:**
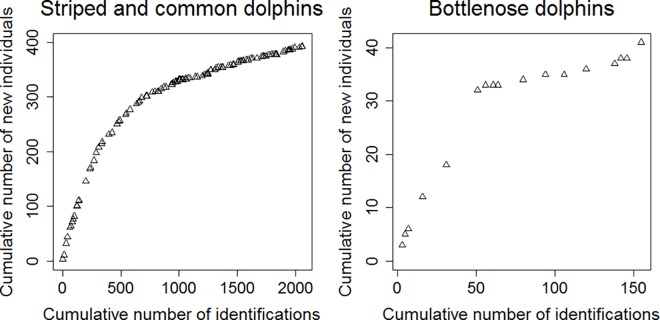
Rate of discovery of new D1 individuals over time for the two photo-identification datasets.

For striped and common dolphins, a total of 23,995 high quality (Q2 and Q3) photographs were analyzed, leading to the identification of 393 D1 individuals. Of those, 72 individuals (18%) were photographed in only one year, 72 (18%) in two years, 90 (23%) in three years, 96 (24%) in four years and 63 (16%) in each of the five years. The number of newly identified individuals (“rate of discovery”) decreased over time (Fig 2). At the end of the fifth year of photo-identification, more than 90% of the individuals (either striped or common dolphins) had already been photographically captured in previous years (Table 2).

For bottlenose dolphins, a total of 2,472 high quality photographs were analyzed, resulting in 41 D1 individuals identified. Of these, 13 (32%) were sighted in only one year, 10 (24%) in two years, 11 (27%) in three years, 7 (17%) in four years, and no individuals in all the five years.

### Model selection and abundance of marked individuals

For the striped and common dolphin dataset the overall goodness of fit test was significant (χ ^2^ = 18.03; *p* < 0.05, df = 8). The lack of fit was mainly due to the significance of TEST 3.Sm that has no clear biological interpretation [69]. We therefore corrected our estimates for a variance inflation factor ĉ = 18.03/8 = 2.25 to accommodate for the lack of fit. The best-fitting model included a constant apparent survival rate, monthly and yearly variation in capture probability, and no temporary emigration between years. No model was within 2ΔQAICc from the best-fitting model (Table 3). Models including individual heterogeneity of capture probability were not supported (data for ΔQAICc > 47 are in S3 Table). Model parameter estimates are shown in Table 4.

**Table 3:** First ten models applied to the striped and common dolphin dataset, ranked by lowest QAICc, number of parameters (n par) and difference in QAICc scores (ΔAICc). QAICc weights indicate strength of evidence for a given model. S(year) = yearly variation in apparent survival; S(.) = no variation in apparent survival; p(year.month) = yearly and monthly variation in capture probability; p(month) = monthly variation in capture probability.

Model	n par	QAICc	ΔQAICc	QAICc weight
S(.)p(year.month) no emigration	25	-1,737.89	0.00	0.57
S(.)p(year.month) random emigration	26	-1,735.82	2.06	0.20
S(year)p(year.month) no emigration	28	-1,734.57	3.32	0.11
S(.)p(year.month) Markovian emigration	27	-1,733.76	4.13	0.07
S(year)p(year.month) random emigration	29	-1,732.49	5.39	0.04
S(year)p(year.month) Markovian emigration	30	-1,730.42	7.46	0.01
S(.)p(month) no emigration	10	-1,695.78	42.11	0.00
S(.)p(month) random emigration	11	-1,693.75	44.14	0.00
S(year)P(month) random emigration	14	-1,692.94	44.94	0.00
S(.)p(month) Markovian emigration	12	-1,691.72	46.16	0.00

**Table 4:** Parameter estimates (with 95% confidence interval) for best model for the striped and common dolphin dataset; n = number of photo-identified individuals (D1), θ = mark ratio, N marked = estimated abundance of D1 individuals; CV = coefficient of variation, S = apparent survival probability; p = capture probability.

Primary occasion	n	θ	N marked	CV	S	Secondaryoccasion	P
Summer 2011	215	0.24	379 (315–455)	0.09	0.94 (0.92–0.96)	May	0.22 (0.16–0.28)
						June	0.11 (0.08–0.15)
						July	0.38 (0.30–0.47)
Summer 2012	246	0.26	327 (301–354)	0.04	0.94 (0.92–0.96)	June	0.21 (0.17–0.26)
						July	0.25 (0.21–0.30)
						August	0.34 (0.28–0.39)
						September	0.37 (0.32–0.43)
Summer 2013	234	0.27	318 (292–346)	0.04	0.94 (0.92–0.96)	June	0.32 (0.27–0.37)
						July	0.32 (0.27–0.37)
						August	0.35 (0.29–0.40)
						September	0.12 (0.09–0.16)
Summer 2014	269	0.25	356 (330–385)	0.04	0.94 (0.92–0.96)	June	0.18 (0.14–0.23)
						July	0.20 (0.16–0.24)
						August	0.42 (0.36–0.47)
						September	0.36 (0.31–0.42)
Summer 2015	221	0.23	350 (315–389)	0.05		June	0.16 (0.13–0.21)
						July	0.13 (0.10–0.17)
						August	0.32 (0.27–0.37)
						September	0.28 (0.23–0.33)

For the bottlenose dolphin dataset, there was no sign of lack of fit (χ^2^ = 1.62, *p* > 0.5, df = 7). The best-fitting model had daily variation in capture probability, constant survival, and random temporary emigration between years (Table 5). Six more models were within 2ΔAICc from the best-fitting model (Table 5), and therefore we resorted to model averaging considering the first six models in Table 6 and obtained the model-averaged estimates listed in Table 6. The model-averaged temporary emigration probability (Ƴ’) was estimated to be 0.16 (SE = 0.13). Models with ΔAICc > 4.99 are shown in S4 Table.

**Table 5:** First ten models applied to the bottlenose dolphin dataset ranked by lowest AICc, number of parameters (n par) and difference in AICc scores (ΔAICc). AICc weights indicate strength of evidence for a given model. S(.) = no variation in apparent survival; p(day) = daily variation in capture probability; p(year) = yearly variation in capture probability; p(day.year) = daily and yearly variation in capture probability.

Model	n par	AICc	ΔAICc	AICc weight
S(.)p(day) random emigration	11	170.14	0.00	0.24
S(.)p(day.year) random emigration	24	171.05	0.91	0.15
S(.)p(day.year) no emigration	23	171.26	1.12	0.14
S(.)p(day) Markovian emigration	12	171.98	1.83	0.10
S(year)p(year) random emigration	14	172.03	1.89	0.09
S(.)p(year) no emigration	11	172.12	1.98	0.09
S(.)p(year) random emigration	12	172.78	2.64	0.07
S(.)p(day.year) Markovian emigration	25	173.85	3.71	0.04
S(.)p(year) Markovian emigration	15	174.34	4.20	0.03
S(.)p(year) Markovian emigration	13	175.13	4.99	0.02

**Table 6:** Parameter estimates (with 95% confidence interval) for best model for the bottlenose dolphin dataset; n = number of photo-identified individuals (D1), θ = mark ratio, N marked = estimated abundance of D1 individuals; CV = coefficient of variation, S = apparent survival probability; p = capture probability.

Primary occasion	n	θ	N marked	CV	S	Secondary occasion	p
June/July 2011	5	0.65	10 (3–33)	0.69	0.86 (0.63–0.96)	15 June	0.27 (0.08–0.63)
						20 June	0.26 (0.07–0.60)
						08 July	0.30 (0.08–0.68)
July 2012	30	0.78	39 (31–50)	0.13	0.86 (0.70–0.94)	08 July	0.29 (0.17–0.45)
						10 July	0.36 (0.23–0.52)
						11 July	0.45 (0.28–0.64)
July 2013	20	0.77	24 (17–34)	0.18	0.87 (0.64–0.96)	08 July	0.26 (0.13–0.44)
						09 July	0.27 (0.13–0.48)
						10 July	0.29 (0.09–0.64)
						11 July	0.62 (0.28–0.87)
June 2014	25	0.69	26 (23–29)	0.06	0.82 (0.42–0.96)	11 June	0.42 (0.19–0.69)
						13 June	0.40 (0.22–0.62)
						16 June	0.48 (0.30–0.67)
						17 June	0.70 (0.49–0.85)
July 2015	13	0.62	22 (11–42)	0.35		06 July	0.23 (0.09–0.47)
						13 July	0.25 (0.09–0.50)
						14 July	0.35 (0.18–0.58)

### Mark ratio, total abundance and species proportions

All estimates are reported with their 95% confidence interval between parentheses. For striped, common and intermediate dolphins the mark ratios are reported in Table 4. Using these proportions, the cumulative total population abundance was 1,593 (1,257–2,018) in 2011, 1,272 (1,022–1,583) in 2012, 1,199 (958–1,500) in 2013, 1,439 (1,168–1,774) in 2014 and 1,535 (1,218–1,933) in 2015. We applied to these cumulative estimates the estimated species proportions of the population: 0.944 (0.910–0.981) for striped dolphins, 0.017 (0.007–0.025) for common dolphins and 0.039 (0.010–0.066) for intermediate animals. Taking these proportions into account, we obtained separate abundance estimates for striped, common and intermediate dolphins (Table 7).

**Table 7:** Total abundance estimate (N tot) and 95% CI for the three species and intermediate animals.

	Striped dolphins			Common dolphins			Intermediate dolphins			Bottlenose dolphins		
Year	N tot	95% CI	CV	N tot	95% CI	CV	N tot	95% CI	CV	N tot	95% CI	CV
2011	1,506	(1,108–2,045)	0.16	25	(14–47)	0.32	62	(29–132)	0.40	15	(5–42)	0.56
2012	1,202	(884–1,635)	0.16	20	(11–37)	0.32	49	(23–105)	0.40	50	(38–66)	0.14
2013	1,133	(836–1,537)	0.16	19	(10–35)	0.32	47	(22–99)	0.40	31	(21–44)	0.19
2014	1,361	(1,028–1,800)	0.14	23	(12–42)	0.32	56	(27–118)	0.39	38	(29–49)	0.14
2015	1,451	(1,060–1,985)	0.16	24	(13–45)	0.32	60	(28–127)	0.39	35	(19–63)	0.31

For bottlenose dolphins, the mark ratios are reported in Table 6. After applying these corrections, we obtained total population abundances shown in Table 7. The estimate obtained for 2011 was considered unreliable due to small sample size (only 5 individuals and 1 recapture).

### Trends in abundance

**Fig 3:**
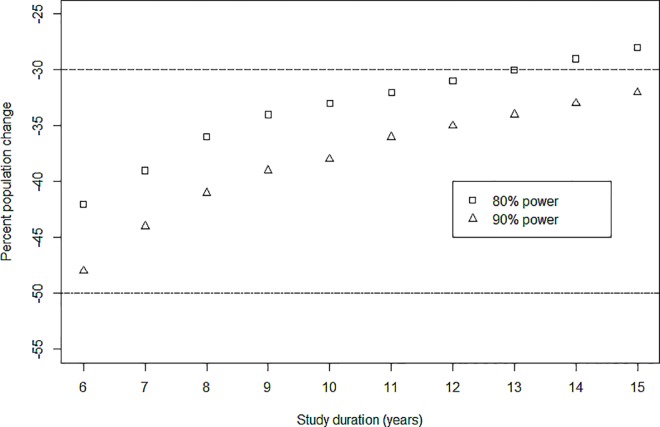
Power analysis for striped dolphins. Percent population changes that we were able to detect for striped dolphins, with a power of 0.8 (squares) and 0.9 (triangles), as a function of the duration of the study (yearly samples). The dashed lines indicate a percent population change of respectively -50 and -30.

For striped dolphins the average CV of the estimates was 0.155. The minimum rate of population decline detectable with a statistical power of 0.8 was an overall decrease of 47% (Fig 3). Such decrease was not detected in a linear regression (see S1 Fig). Detecting a slighter decrease in abundance (i.e. 30%) with a power of 0.8 would require an additional 8 years of monitoring (i.e. a total of 13 years of monitoring; Fig 3).

For common dolphins the average CV of the estimates was 0.320. The minimum rate of population decline detectable in 5 years with a 0.8 statistical power was an overall decrease of 80%.

For bottlenose dolphins the average CV of the estimates was 0.192. The minimum rate of population decline detectable with 0.8 statistical power (based on 4 yearly samples, because 2011 estimate was discarded) was an overall decrease of 67%. Such decrease was not detected in a linear regression (see S2 Fig).

## Discussion

### Temporary emigration patterns

We tested a range of models on different dolphin datasets, to account for presence and absence of temporary emigration. The best models supported previous knowledge about movement patterns of the three species in the Gulf of Corinth. For striped and common dolphins, absence of temporary emigration was the most likely scenario indicating that no movements occurred outside of the study area between primary sampling occasions. However, this result refers only to the intervals between primary sampling occasions and cannot be extended to other time frames.

Random temporary emigration was instead detected for bottlenose dolphins and each individual had about a 16% probability of being outside the study area during a primary occasion. Such result is in agreement with information reported by [26]. The authors compared photo-identification catalogs from the Gulf of Corinth and other areas in the Ionian Sea, and found that 9 of 31 individuals identified in the Gulf were also photographed in areas up to 265 km apart.

Differences in ranging patterns between odontocete populations have been related to availability of food resources [74, 75, 76]. In the Gulf of Corinth, [4] inferred a relatively high abundance of pelagic prey resources based on the abundance of pelagic predators, and suggested that availability of such prey may sustain a year-round resident population of striped and common dolphins. Conversely, scarce and patchily-distributed demersal prey may prompt movements of bottlenose dolphins to distant areas [26, 74, 75, 76]. An assessment of the status of fish and cephalopod stocks in the area, lacking at the moment, would help clarify this point.

### Mark rate

For striped and common dolphins the mark rate could be considered low (an average of 0.25 over the 5 years) if compared to other photo-identification studies on dolphin species (mostly *Tursiops* sp) in which the mark rate is often > 0.5 e.g. [38, 40, 77, 78]. However, the uncertainty associated to the proportion of unmarked animals is taken into account in the total population estimate because the lower is the mark rate, the higher is the variance [34]. [79] estimated the abundance of a population of Hector’s dolphins (*Cephalorhynchus hectori*) in New Zealand, based on a mark rate of 0.104, and obtained an estimate which was consistent with a parallel and independent one obtained through distance sampling by the same authors, indicating that capture-recapture methods can produce accurate estimates even with a low percentage of marked animals. Furthermore, increasing the mark ratio by including less distinctive animals (D2) is risky because it could bias the abundance estimate upwards as recapture probabilities would be lowered [35]. We therefore consider the estimates obtained with only highly distinctive individuals (D1) as more reliable and conservative, and less likely to invalidate CR assumptions of correct mark recognition and homogeneous capture probability.

### Abundance estimates

Our point estimates of striped dolphins are higher than the one obtained by [6] using CR methods. We urge caution in comparing the results of the two studies because the sampling and analytical strategies implemented simply cannot be compared. [6] covered only the central part of the Gulf and sampled considerably fewer groups of dolphins (23 in a single year of sampling, versus our average of 85 groups per year across five years of sampling). The differences in the estimates likely reflect the different coverage and sample sizes [34, 80, 81, 82] and imply that sampling by [6] was insufficient as suggested by [83].

Common dolphins occur in critically low numbers (see Table 7). While historical information is lacking for this area, a steep decline of this species has been documented for the larger Mediterranean region, including in portions of the Ionian Sea, due to prey depletion by fisheries and incidental mortality in fishing gear [11, 84]. An additional threat in the Gulf of Corinth may be hybridization with striped dolphins. Hybridization among Delphininae is not rare, with many intergeneric and intrageneric pairs of species able to produce viable hybrid offsprings, and in at least some cases viable backcrosses [85]. Hybridization is a relatively unexplored cause of extinction, especially for small populations that mix with more abundant ones [86, 87]. In this context, our estimate of intermediate dolphins (most likely hybrids) is an important baseline to monitor hybridization dynamics over time and its impact on the viability of the two species.

An average of 38 (32–46) bottlenose dolphins were found to occur the Gulf of Corinth from 2012 to 2015. Given bottlenose dolphin movement patterns in and out of the Gulf, our yearly estimates should be interpreted strictly as the number of individuals using the area during the primary occasions and inter-annual variability is unlikely to reflect fluctuations in population size [39]. In other areas, seasonal variations in the abundance of bottlenose dolphins have been related to temporal shift in habitat use, due to factors including reproduction [52], disturbance, and prey availability [23, 40]. Comparisons with photo-identification catalogs from other areas in the Ionian Sea (and beyond) as well as genetic analyses would be needed to investigate population structure [88].

### Apparent survival rates

The most parsimonious model for striped and common dolphins had constant apparent survival. Apparent survival reflects true survival if there is no permanent emigration from the population [41]. Genetic and distribution data would support such hypothesis for striped and common dolphins in the Gulf of Corinth, and our estimate of annual apparent survival is likely representative of true survival. Since striped, common and intermediate individuals were pooled together in the CR analyses, we could not obtain separate survival estimates, nor could we discriminate between adults and juveniles. However, our survival estimate is largely representative of striped dolphins (representing 94.4% of the assessed population). To our knowledge, this is the first annual survival estimate for any striped dolphin population, worldwide. The estimated survival of 0.94 (0.92–0.96, SE = 0.01) is similar to annual survival estimated for spinner dolphins *Stenella longirostris* in Hawaii, i.e. 0.97 (SE = 0.05; [41]) and it is high, as expected, for slowly reproducing mammals whose life span is longer than the study duration [53, 89].

Average apparent survival for bottlenose dolphins was 0.85 (0.76–0.95, SE = 0.04). Such estimate is lower than those obtained for adult individuals of the same species in other areas. For instance, a study conducted in the northern Adriatic Sea found adult survival ranging between 0.825 ± 0.054 SE and 0.938 ± 0.042 SE [90]. Another study in the Azores found 0.97 ± 0.03 SE [38] and a study in New Zealand 0.94 (0.92–0.95; [91]). However, our estimate is consistent with estimated survival of juveniles in the Azores which is 0.815 ± 0.083 [38]. Two factors may bias our survival estimates downwards. First, the presence of subadults that have lower survival rates than adults [67]. Second, the occurrence of transient individuals [26] that may cause an underestimation of apparent survival [92]. This phenomenon was not detected by a TEST 3.SR performed on our dataset, likely due to the low power of such test with reduced sample sizes [93].

### Conservation and management implications

The abundance and survival estimates produced in this study can be used for the evaluation of these populations’ extinction risks trough Population Viability Analysis [24, 94, 95, 96, 97] and, ideally, for conservation status assessment following IUCN criteria for regional populations [24, 98]. A challenge is to incorporate the effect of hybridization on demographic rates [87].

Even though striped dolphins are the most abundant species, their estimated abundance is still well below the threshold that would qualify this local population as Vulnerable (10,000 mature individuals) or Endangered (2,500), if coupled with a continuous population decline of respectively 10 or 20% [98, 99]. For striped dolphins our monitoring showed sufficient power to detect a precipitous decline of 50% in the population during the entire study. Such decline was not detected (see S1 Fig). Continuation of monitoring appears essential to assess the conservation status of this species.

Our results can be used to assess the most cost-effective management strategy to detect population trends [100]. Nonetheless, if management measures are to be taken only if a decline is detected, many additional years are needed before this actually happens (for example 8 more years to detect a 30% decline). Interestingly, the relatively high numbers of striped dolphins would allow us to use a combination of power and population viability analyses [101] to assess the benefits and costs of implementing management actions before a decline is detected (as prescribed by a precautionary approach) or after (traditional approach).

Power to detect trends in abundance decreases as a population becomes smaller [2]: for bottlenose and especially common dolphins, the power to detect trends is exceedingly low. As a consequence, also considering low population numbers, conservation measures should be put in action regardless of the detection of a decline [2].

## Conclusions

In summary, we successfully applied a Robust Design analytic framework to a challenging scenario with different odontocete populations characterized by distinct movement patterns and mixed-species groups. We contributed revised baseline data of abundance and survival for striped dolphins, common dolphins and possible hybrids between the two species in the Gulf of Corinth. We provided the first estimate of survival, abundance and temporary emigration for bottlenose dolphins in this part of Greece. Finally, we illustrated the importance and the power of long-term monitoring to provide baselines for future conservation and management of cetaceans in this area.

## Supporting Information

S1 Fig Estimates of abundance of striped dolphins over the 5 years of the study. The line represents the regression of abundance over time. The determination coefficient (R squared) and the significance level are reported.
(JPEG)

S2 Fig Estimates of abundance of bottlenose dolphins. Year 2011 is omitted from the regression because the estimate was judged non-reliable (see Results section). The line represents the regression of abundance over time. The determination coefficient (R squared) and the significance level are reported.
(JPEG)

S1 File Dataset used for striped and common dolphin CR analysis. (TXT)

S2 File Dataset used for bottlenose dolphin CR analysis. (TXT)

S3 File Example of R script used for performing CR analysis on both datasets. (TXT)

S1 Table List of survey days used to build the capture-recapture matrix. The whole study area was covered at least once per secondary occasion (months).
(PDF)

S2 Table Total number of sampling days with bottlenose dolphin encounters. The days used to build the capture-recapture matrix are highlighted in bold.
(PDF)

S3 Table Robust design models applied to the striped and common dolphin dataset. The models are ranked by lowest QAICc, number of parameters (npar) and difference in QAICc scores (ΔAICc). QAICc weights indicate strength of evidence for a given model. S(year) = yearly variation in apparent survival; S(.) = no variation in apparent survival; p(year.month) = yearly and monthly variation in capture probability; p(month) = monthly variation in capture probability, p(mixture) = individual heterogeneity in capture probability.
(PDF)

S4 Table Robust design models applied to the bottlenose dolphin dataset. The models are ranked by lowest QAICc, number of parameters (npar) and difference in QAICc scores (ΔAICc). QAICc weights indicate strength of evidence for a given model. S(year) = yearly variation in apparent survival; S(.) = no variation in apparent survival; p(year.month) = yearly and monthly variation in capture probability; p(month) = monthly variation in capture probability, p(mixture) = individual heterogeneity in capture probability.
(PDF)
