# Supplementary figures and images for: A Robust Design Capture-Recapture Analysis of Abundance, Survival and Temporary Emigration of Three Odontocete Species in the Gulf of Corinth, Greece
Source: PLoS One. 2016 Dec 7;11(12):e0166650. doi: 10.1371/journal.pone.0166650 (PMC5142793; doi:10.1371/journal.pone.0166650)

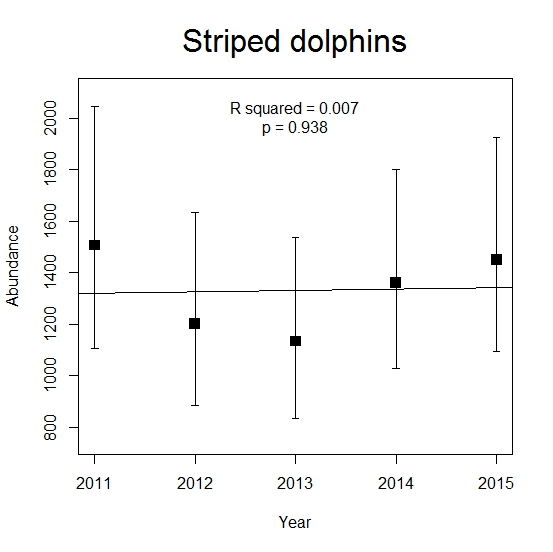

Supplement: S1 Fig — The line represents the regression of abundance over time. The determination coefficient (R squared) and the significance level are reported. (JPEG) [file pone.0166650.s001.jpeg]

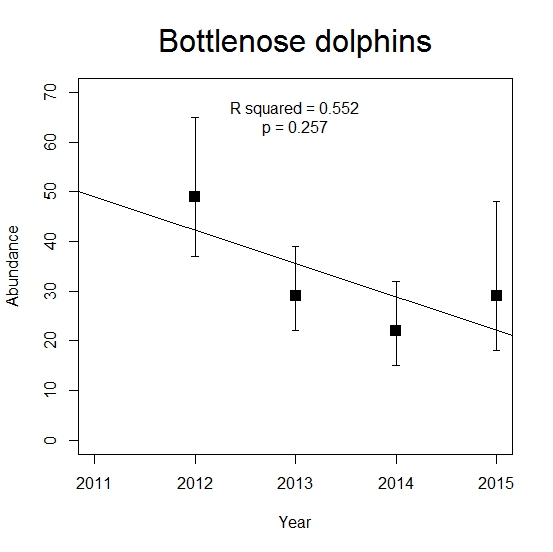

Supplement: S2 Fig — Year 2011 is omitted from the regression because the estimate was judged non-reliable (see Results section). The line represents the regression of abundance over time. The determination coefficient (R squared) and the significance level are reported. (JPEG) [file pone.0166650.s002.jpeg]
