# Supplementary material for: A Robust Design Capture-Recapture Analysis of Abundance, Survival and Temporary Emigration of Three Odontocete Species in the Gulf of Corinth, Greece
Source: PLoS One. 2016 Dec 7;11(12):e0166650. doi: 10.1371/journal.pone.0166650 (PMC5142793; doi:10.1371/journal.pone.0166650)
Supplement: S1 Table — The whole study area was covered at least once per secondary occasion (months). (PDF) [file pone.0166650.s006.pdf]

S1 Table. List of survey days used to build the capture-recapture matrix. The whole study area was covered at least once per secondary occasion (months).

	2011	2012	2013	2014	2015
Secondary 1 (sampling days)	12, 13, 14, 20, 21, 23, 28, 29, 30 May	7, 8, 9, 10 June	6, 7, 8, 17, 18, 22, 23 June	7, 12, 16, 18, 23, 29, June	4, 9, 23, 26, 29, 30 June
Secondary 2 (sampling days)	14, 15, 22, 24 June	8, 10, 11, 15 July	2, 3, 4, 5, 13, 15, 16, 20, 28, 29, 30 July	7, 8, 16, 17, 26 July	7, 8, 9, 13, 24 July
Secondary 3 (sampling days)	2, 7, 8, 9, 13, 14, 16 July	5, 7, 16, 23, 25 August	10, 12, 13, 19, 24 August	5, 10, 11, 12, 14, 20, 21, 26, 28, 31 August	1, 3, 5, 13, 15, 23, 24, 26, 29 August
Secondary 4 (sampling days)		12, 17, 19, 22, 24, 27, 28 September	2, 7, 9 September	8, 9, 10, 11, 14, 20 September	1, 2, 11, 13, 15, 18, 22, 26 September
