# Supplementary material for: A Robust Design Capture-Recapture Analysis of Abundance, Survival and Temporary Emigration of Three Odontocete Species in the Gulf of Corinth, Greece
Source: PLoS One. 2016 Dec 7;11(12):e0166650. doi: 10.1371/journal.pone.0166650 (PMC5142793; doi:10.1371/journal.pone.0166650)
Supplement: S2 Table — The days used to build the capture-recapture matrix are highlighted in bold. (PDF) [file pone.0166650.s007.pdf]

S2 Table. Total number of sampling days with bottlenose dolphin encounters. The days used to build the capture-recapture matrix are highlighted in bold.

2011	2012	2013	2014	2015
12 May	8 June	6 May	**11 June**	1 June
29 May	**8 July**	18 May	**13 June**	1 July
**15 June**	**10 July**	18 June	**16 June**	**6 July**
**20 June**	**11 July**	19 June	**17 June**	**13 July**
**8 July**	6 August	**8 July**	30 June	**14 July**
	7 August	**9 July**	6 July	26 July
	24 September	**10 July**	7 July	16 September
		**11 July**	10 July	9 October
			11 July	
			20 July	
			7 August	
			19 September	
