# Supplementary material for: A Robust Design Capture-Recapture Analysis of Abundance, Survival and Temporary Emigration of Three Odontocete Species in the Gulf of Corinth, Greece
Source: PLoS One. 2016 Dec 7;11(12):e0166650. doi: 10.1371/journal.pone.0166650 (PMC5142793; doi:10.1371/journal.pone.0166650)
Supplement: S3 Table — The models are ranked by lowest QAICc, number of parameters (npar) and difference in QAICc scores (ΔAICc). QAICc weights indicate strength of evidence for a given model. S(year) = yearly variation in apparent survival; S(.) = no variation in apparent survival; p(year.month) = yearly and monthly variation in capture probability; p(month) = monthly variation in capture probability, p(mixture) = individual heterogeneity in capture probability. (PDF) [file pone.0166650.s008.pdf]

**S3 Table**. Robust design models applied to the striped and common dolphin dataset. The models are ranked by lowest QAICc, number of parameters (npar) and difference in QAICc scores (ΔAICc). QAICc weights indicate strength of evidence for a given model. S(year) = yearly variation in apparent survival; S(.) = no variation in apparent survival; p(year.month) = yearly and monthly variation in capture probability; p(month) = monthly variation in capture probability, p(mixture) = individual heterogeneity in capture probability.

| Model | npar | QAICc | DeltaQAICc | weight |
|---|---|---|---|---|
| S(.)p(year.month) no emigration | 25 | -1,737.89 | 0.00 | 0.57 |
| S(.)p(year.month) random emigration | 26 | -1,735.82 | 2.06 | 0.20 |
| S(year)p(year.month) no emigration | 28 | -1,734.57 | 3.32 | 0.11 |
| S(.)p(year.month) Markovian emigration | 27 | -1,733.76 | 4.13 | 0.07 |
| S(year)p(year.month) random emigration | 29 | -1,732.49 | 5.39 | 0.04 |
| S(year)p(year.month) Markovian emigration | 30 | -1,730.42 | 7.46 | 0.01 |
| S(.)p(month) no emigration | 10 | -1,695.78 | 42.11 | 0.00 |
| S(.)p(month) random emigration | 11 | -1,693.75 | 44.14 | 0.00 |
| S(year)p(month) random emigration | 14 | -1,692.94 | 44.94 | 0.00 |
| S(.)p(month) Markovian emigration | 12 | -1,691.72 | 46.16 | 0.00 |
| S(year)p(month) Markovian emigration | 15 | -1,690.91 | 46.98 | 0.00 |
| S(.)p(year) no emigration | 11 | -1,642.70 | 95.19 | 0.00 |
| S(.)p(year) random emigration | 12 | -1,640.67 | 97.21 | 0.00 |
| S(.)p(.) no emigration | 7 | -1,640.32 | 97.57 | 0.00 |
| S(year)p(year) no emigration | 14 | -1,640.04 | 97.85 | 0.00 |
| S(year) p(.) no emigration | 10 | -1,639.25 | 98.63 | 0.00 |
| S(year)p(.) no emigration | 10 | -1,639.25 | 98.63 | 0.00 |
| S(.)p(year) Markovian emigration | 13 | -1,638.64 | 99.25 | 0.00 |
| S(.)p(.) random emigration | 8 | -1,638.30 | 99.59 | 0.00 |
| S(year)p(year) random emigration | 15 | -1,638.00 | 99.88 | 0.00 |
| S(year)p(.) random emigration | 11 | -1,637.23 | 100.66 | 0.00 |
| S(.)p(~1) Markovian emigration | 9 | -1,636.28 | 101.61 | 0.00 |
| S(.)p(mixture) no emigration | 9 | -1,636.28 | 101.61 | 0.00 |
| S(year)p(year) Markovian emigration | 16 | -1,635.97 | 101.92 | 0.00 |
| S(year)p(~1) Markovian emigration | 12 | -1,635.20 | 102.69 | 0.00 |
| S(year) p(~mixture) no emigration | 12 | -1,635.20 | 102.69 | 0.00 |
| S(.)p(mixture) random emigration | 10 | -1,634.25 | 103.63 | 0.00 |
| S(year)p(mixture) random emigration | 13 | -1,633.17 | 104.72 | 0.00 |
| S(.)p(mixture) Markovian emigration | 11 | -1,632.23 | 105.66 | 0.00 |
| S(year)p(mixture) Markovian emigration | 14 | -1,631.13 | 106.75 | 0.00 |
