# Supplementary material for: A Robust Design Capture-Recapture Analysis of Abundance, Survival and Temporary Emigration of Three Odontocete Species in the Gulf of Corinth, Greece
Source: PLoS One. 2016 Dec 7;11(12):e0166650. doi: 10.1371/journal.pone.0166650 (PMC5142793; doi:10.1371/journal.pone.0166650)
Supplement: S4 Table — The models are ranked by lowest QAICc, number of parameters (npar) and difference in QAICc scores (ΔAICc). QAICc weights indicate strength of evidence for a given model. S(year) = yearly variation in apparent survival; S(.) = no variation in apparent survival; p(year.month) = yearly and monthly variation in capture probability; p(month) = monthly variation in capture probability, p(mixture) = individual heterogeneity in capture probability. (PDF) [file pone.0166650.s009.pdf]

S4 Table. Robust design models applied to the bottlenose dolphin dataset. The models are ranked by lowest QAICc, number of parameters (npar) and difference in QAICc scores (ΔAICc). QAICc weights indicate strength of evidence for a given model. S(year) = yearly variation in apparent survival; S(.) = no variation in apparent survival; p(year.month) = yearly and monthly variation in capture probability; p(month) = monthly variation in capture probability, p(mixture) = individual heterogeneity in capture probability.

Model	npar	QAICc	DeltaAICc	weight
S(.)p(day) random emigration	11	170.14	0.00	0.24
S(.)p(day.year) random emigration	24	171.05	0.91	0.15
S(.)p(day.year) no emigration	23	171.26	1.12	0.14
S(.)p(day) Markovian emigration	12	171.98	1.83	0.10
S(year)p(day) random emigration	14	172.03	1.89	0.09
S(.)p(year) no emigration	11	172.12	1.98	0.09
S(.)p(year) random emigration	12	172.78	2.64	0.07
S(.)p(day.year) Markovian emigration	25	173.85	3.71	0.04
S(year)p(day) Markovian emigration	15	174.34	4.20	0.03
S(.)p(year) Markovian emigration	13	175.13	4.99	0.02
S(year)p(year) no emigration	14	177.22	7.08	0.01
S(year)p(day.year) no emigration	26	177.42	7.28	0.01
S(.)p(day) no emigration	10	177.83	7.69	0.01
S(year)p(day.year) random emigration	27	178.07	7.93	0.00
S(year)p(year) random emigration	15	178.51	8.37	0.00
S(year)p(year) Markovian emigration	16	180.97	10.83	0.00
S(year)p(day.year) Markovian emigration	28	181.03	10.89	0.00
S(year)p(.) random emigration	11	187.35	17.21	0.00
S(.)p(.) random emigration	8	188.60	18.46	0.00
S(year)p(.) no emigration	10	189.60	19.46	0.00
S(year)p(.) no emigration	10	189.60	19.46	0.00
S(year)p(.) Markovian emigration	12	189.67	19.53	0.00
S(year)p(mixture) no emigration	12	189.93	19.79	0.00
S(.)p(.) Markovian emigration	9	190.52	20.38	0.00
S(.)p(mixture) no emigration	9	191.04	20.90	0.00
S(year)p(mixture) random emigration	13	191.05	20.91	0.00
S(.)p(mixture) random emigration	10	191.87	21.73	0.00
S(.)p(.) no emigration	7	192.73	22.59	0.00
S(year) p(mixture) Markovian emigration	14	193.43	23.29	0.00
S(.)p(mixture) Markovian emigration	11	193.88	23.74	0.00
